# Computational Approaches and Challenges in Spatial Transcriptomics

**DOI:** 10.1016/j.gpb.2022.10.001

**Published:** 2022-10-14

**Authors:** Shuangsang Fang, Bichao Chen, Yong Zhang, Haixi Sun, Longqi Liu, Shiping Liu, Yuxiang Li, Xun Xu

**Affiliations:** 1 BGI-Shenzhen, Shenzhen 518083, China; 2 BGI-Beijing, Beijing 100101, China; 3 Guangdong Bigdata Engineering Technology Research Center for Life Sciences, Shenzhen 518083, China

**Keywords:** Spatial transcriptomics, Computational approach, Data quality, Data interpretation, Multi-omics integration

## Abstract

The development of **spatial transcriptomics** (ST) technologies has transformed genetic research from a single-cell data level to a two-dimensional spatial coordinate system and facilitated the study of the composition and function of various cell subsets in different environments and organs. The large-scale data generated by these ST technologies, which contain spatial gene expression information, have elicited the need for spatially resolved approaches to meet the requirements of computational and biological **data interpretation**. These requirements include dealing with the explosive growth of data to determine the cell-level and gene-level expression, correcting the inner batch effect and loss of expression to improve the **data quality**, conducting efficient interpretation and in-depth knowledge mining both at the single-cell and tissue-wide levels, and conducting **multi-omics integration** analysis to provide an extensible framework toward the in-depth understanding of biological processes. However, algorithms designed specifically for ST technologies to meet these requirements are still in their infancy. Here, we review **computational approaches** to these problems in light of corresponding issues and challenges, and present forward-looking insights into algorithm development.

## Introduction

The cellular and molecular characteristics of multicellular organisms are influenced by the surrounding cell and tissue environments. Therefore, determining the spatial location of cells is important for the study of cell differentiation, cell communication, tissue structure, and tissue microenvironment. Breakthroughs in a number of new spatial transcriptomics (ST) technologies have made it possible to accurately locate cells. *In situ* hybridization (ISH)-based technologies (*e.g.*, smFISH [1], MERFISH [2], and seqFISH [3], [4]) hybridize the targeted RNA sequences with pre-designed probes and use spectral barcodes or sequential imaging technologies to capture fluorescent signals for transcript identification. However, these technologies lack the capacity to discover new transcriptomes and isoforms. *In situ* sequencing (ISS)-based technologies (*e.g.*, FISSEQ [5] and STARmap [6]) use micron- or nanometer-sized DNA balls to enhance RNA signals to achieve ISS, but can only capture a limited number of genes. In addition, ISH-based and ISS-based technologies require a highly sensitive single-molecule fluorescence imaging system, complex repeated imaging, and further complex image analysis processes. Recently, barcode-based ST technologies have been used to label spatial locations by capturing sequences *in situ* and performing transcriptomics sequencing after elution to overcome the limitations of direct imaging. 10X Visium (spatial resolution: 55 μm), Slide-seq (spatial resolution: 10 μm) [7], and high-definition ST (HDST; spatial resolution: 2 μm) [8] measure RNA expression at the capture location (referred to as the spot) with improved spatial resolution. DBiT-seq [9] uses parallel microfluidic channels to crossflow two sets of barcodes to the tissue surface and ligate them *in situ* to obtain their two-dimensional (2D) coordinates. Digital Spatial Profiling (DSP) [10] uses light photocleaving to release photocleavable oligonucleotides in multiple regions of interest to achieve *in situ* detection of protein and gene information on frozen or paraffin-embedded tissue sections by sequencing. Seq-Scope [11] uses solid-phase amplification to improve the capture efficiency with dramatically improved spatial resolution. The new DNA nanoball (DNB)-based technology, Stereo-seq [12], can achieve a higher resolution and a larger field of view than all the methods mentioned above and profile samples at the size of whole late-stage mouse embryos. For a more comprehensive review of ST technologies, we refer readers to references [13], [14], [15], [16], [17]. The long-term development goals of ST technology are to achieve finer detail at the single-cell level, higher resolution, higher sensitivity, a larger field of view at the microscopic level, and more extensive spatial multi-omics applications.

The development of ST has produced a large amount of valuable spatial data, which have greatly improved the feasibility of interpreting biological mechanisms through omics data, and thus, ST has been applied in many research fields. For example, various methods have been applied to assess the homeostasis and development of healthy tissue. Chen et al. generated the first panoramic transcriptomic atlas of mouse organogenesis and obtained insights into the molecular basis of regional specification, neuronal migration, and differentiation in the developing brain [12]. Crosse et al. spatially characterized the developing hematopoietic stem cell (HSC) niche and identified factors secreted in early human HSC development [18]. ST technologies have also been applied to study tissue composition and function. Hildebrandt et al. delineated a wide range of spatial gene expression patterns in the liver, identifying important effects on liver function, development, and regeneration as well as the potential of these expression patterns for clinical applications [19]. Baccin et al. discovered the bone marrow niche organization at the molecular, cellular, and spatial levels [20]. Investigating the immune microenvironment of cancer and other diseases is another important application of ST technologies. Berglund et al. obtained new insights into gene expression differences between the prostate cancer core and periphery, and these researchers uncovered an “unexplored landscape of heterogeneity” for prostate cancer [21]. Chen et al. untangled the dysregulated cellular network in the vicinity of pathogenic hallmarks of Alzheimer’s disease and other brain diseases [22]. With the increasing number of these kinds of studies being published, the application of ST technologies has expanded substantially.

**Figure 1:**
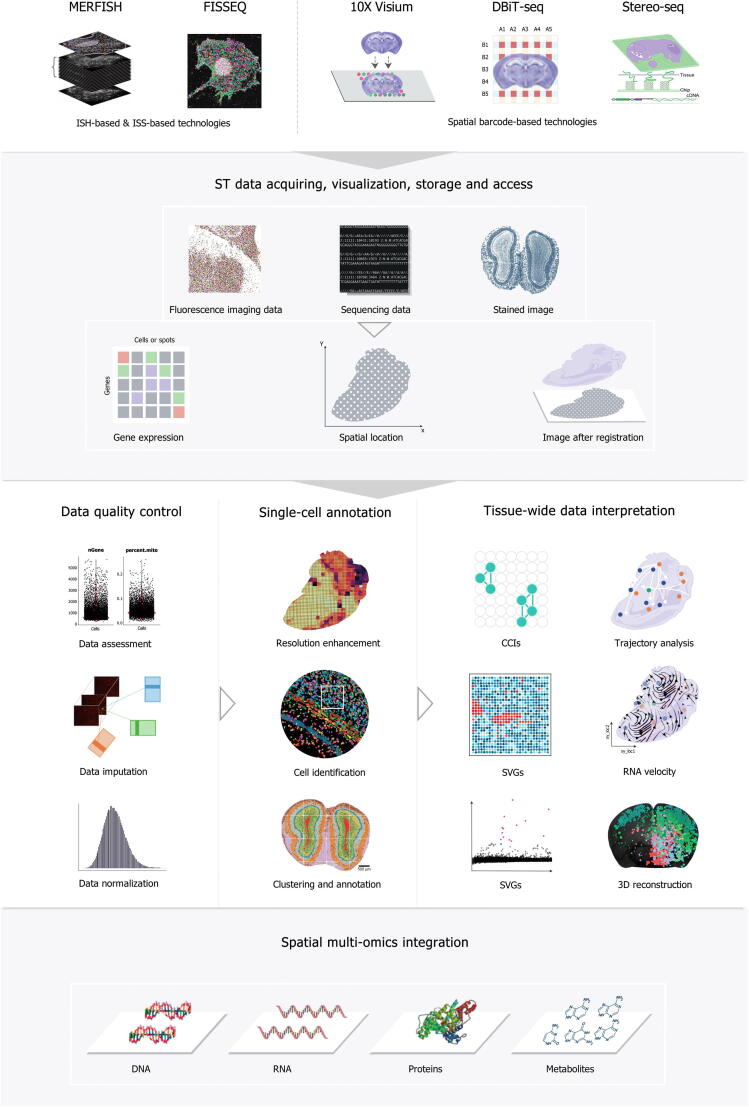
**The main sections inSTdata analysis** Schematic of the five critical sections involved in ST data analysis, including (1) big data acquisition, visualization, storage, and access; (2) data quality control; (3) single cell-level and tissue-level definition; (4) tissue-wide data interpretation; and (5) spatial multi-omics integration. ST, spatial transcriptomics; ISH, *in situ* hybridization; ISS, *in situ* sequencing; SVG, spatial variable gene; CCI, cell–cell interaction; 3D, three-dimensional.

The data generated by ST are an entirely new type and amount of data requiring specialized solutions. The increase in the resolution and the scope of barcode-based ST technologies as well as the throughput of ISH- and ISS-based technologies has led to dramatic increases in data, which brings challenges for data storage and computing. Additionally, the significant differences that exist in the throughput, coverage, and spatial resolution of different technologies make it difficult to develop generalizable algorithms. ST-specific algorithms that utilize spatial information and histology images need to be developed to investigate and interpret data from a spatial perspective. Furthermore, the analysis of ST data is performed at the RNA level and therefore requires a comprehensive knowledge of cell biology, biochemistry, and immunology. To summarize, the explosion in data volume, the utilization of new data features, and the integration of multi-omics knowledge all introduce higher requirements and challenges that must be addressed to analyze ST data effectively. This review focuses on five critical topics related to ST data analysis and interpretation (Figure 1): (1) ST data exploration with regard to data acquiring, visualization, storage, and access; (2) ST data quality control and preprocessing, including data quality assessment, filtering, and quality improvement; (3) single cell- and tissue-level annotations in ST data; (4) tissue-wide ST data interpretation with single or multiple slices in multi-dimensional space; and (5) prospective insights into single-cell and spatial multi-omics. We introduce these topics in the context of current research being done, with consideration of how data files processing pipelines work in general, and while noting the distinctions among the various analysis modules. We also summarize the widely used algorithms and tools (Table 1) and discuss the future development of computing approaches, which might shed light on the advancement of ST data interpretation.

**Table 1:** **Summary of the widely used subset of methods applied inSTdata analysis**

**Method**	**Category**	**Description**	**Advantage**	D**isadvantage**	**ST-specific**	**URL**	**Language**	**Released year**	**Ref.**
stLearn-SME	Data normalization	A method for normalization using neighborhood information and morphological distance	Utilize spatial neighborhood information and morphological distance	Require histology images	Yes	https://github.com/BiomedicalMachineLearning/stLearn	Python	2020	[48]
SCTransform	Data normalization	An R package for normalization and variance stabilization of scRNA-seq data using regularized negative binomial regression	Construct a generalized linear model for each gene instead of a same constant factor	Cannot incorporate spatial and histology information	No	https://github.com/ChristophH/sctransform	R	2019	[54]
gimVI	Data imputation	A method for the imputation of missing genes in ST from scRNA-seq data	Make use of all the genes expressed in scRNA-seq data	Cannot locate important genes in prediction tasks	Yes	https://github.com/YosefLab/scvi-tools	Python	2019	[58]
SpaGE	Data imputation	A method for spatial gene enhancement using scRNA-seq	Predict whole transcriptome expression in their spatial configuration	Use only a certain fraction of the features and cannot take full advantage of the reference	Yes	https://github.com/tabdelaal/SpaGE	Python	2020	[59]
stPlus	Data imputation	A reference-based method for the accurate enhancement of ST	Robust and scalable to datasets of diverse gene detection sensitivity levels and sample sizes	Impute gene expression constrained by the reference (scRNA-seq data)	Yes	https://github.com/xy-chen16/stPlus	Python	2021	[60]
BayesSpace	Spatial clustering; resolution enhancement	A Bayesian statistical model for clustering and resolution enhancement of spatial gene expression experiments	Apply clustering simultaneously with or without resolution enhancement	Cannot incorporate histology information	Yes	https://github.com/edward130603/BayesSpace	R	2020	[62]
SpaGCN	Spatial clustering; SVG	A graph convolutional network to identify spatial domains with coherent gene expression and histology	Incorporate histology information; provide biological interpretations of the identified spatial domains	Cannot account for cell type variations in spatially variable gene detection	Yes	https://github.com/jianhuupenn/SpaGCN	Python	2020	[73]
SEDR	Spatial clustering; trajectory analysis	A deep autoencoder network to generate a low dimensional representation of gene expression by employing unsupervised deep clustering	Generate cell embedding for subsequent tasks	Cannot incorporate histology information	Yes	https://github.com/HzFu/SEDR	Python	2021	[74]
MUSE	Spatial clustering	A multi-view autoencoder neural network to characterize cells and tissue regions by integrating morphological and spatially resolved transcriptional data	Incorporate histology information; discover tissue subpopulations missed by either modality	Require histology information	Yes	https://github.com/AltschulerWu-Lab/MUSE	Python	2022	[71]
BANKSY	Spatial clustering	An algorithm to unify cell type clustering and domain segmentation by constructing a product space of cell and neighborhood transcriptomes	Avoid the pitfall of assuming that cells of the same type or subtype are physically proximal	Cannot incorporate histology information	Yes	https://github.com/prabhakarlab/Banksy_py	Python	2022	[72]
RCTD	Cell-type deconvolution	A statistical model to annotate cell types based on scRNA-seq reference	Normalize ST data and scRNA-seq reference before deconvolution	Ignore the physical distance and the spatial dependency of gene expression	Yes	https://github.com/dmcable/RCTD	R	2020	[75]
SPOTlight	Cell-type deconvolution	A seeded non-negative matrix factorization regression algorithm to infer cell types by integrating scRNA-seq datasets	Achieve high sensitivity and robustness	Infer cell types constrained by the prior information	Yes	https://github.com/MarcElosua/SPOTlight	R	2021	[76]
SpatialDWLS	Cell-type deconvolution	A method to identify the cell types at each location with Giotto and determine the cell type composition using dampened weighted least squares	Enhance specificity for the most likely cell types with a specific location	Cannot account for spatial dependency of gene expression in deconvolution	Yes	https://github.com/rdong08/spatialDWLS_dataset	R	2021	[77]
Cell2location	Cell-type mapping	A Bayesian model that can identifies cell types in ST data and create cellular maps of diverse tissues	Resolve fine-grained cell types in ST data	Cannot account for differences in the noise characteristics	Yes	https://github.com/BayraktarLab/cell2location/	Python	2022	[63]
pciSeq	Cell-type mapping	A probabilistic cell typing algorithm for ST	Utilize probilistic assignment for cell typing to provide a confidence score	Require scRNA-seq data of the tissue of interest	Yes	https://github.com/acycliq/pciSeq	Python	2020	[65]
Baysor	Cell-type mapping	A tool for Bayesian segmentation of ST by considering both cell transcripts and morphological information	Segment cells using molecular position alone or incorporate additional staining information	Require manual processing for better performance on difficult cases	Yes	https://github.com/kharchenkolab/Baysor	Julia	2020	[78]
JSTA	Cell-type mapping	A computational framework to iteratively update cell segmentation by adding cell type probabilities to pixels	Improve the assignment accuracy at pixel level by using existing cell type information	Can only apply to RNA hybridization-based ST	Yes	https://github.com/wollmanlab/JSTA	Python	2021	[66]
SSAM	Cell-type mapping	A cell segmentation-free framework to assign cell types to pixels by using mRNA signals	Scalable to 3D; possible to identify rare cell types	Calculating each gene's density could be a performance concern on non-FISH techniques	Yes	https://github.com/HiDiHlabs/ssam	Python	2019	[64]
CellTrek	Cell-type mapping	A computational method to achieve single-cell spatial mapping through coembedding and metric learning approaches	Achieve single-cell spatial mapping	Require ST spots with relative high cell purities	Yes	https://github.com/navinlabcode/CellTrek	R	2022	[79]
GCNG	CCI	A graph convolutional network to propose novel pairs of extracellular interactions	Incorporate spatial context; identify interactions that are limited to a specific area or specific cell types, or that are related to more complex patterns	Infer CCIs constrained by the prior information	Yes	https://github.com/xiaoyeye/GCNG	Python	2020	[89]
CellPhoneDB	CCI	A novel repository of ligands, receptors, and their interactions which integrates with a statistical framework that predicts enriched cellular interactions	Take multi-subunit protein complexes into consideration	Cannot incorporate spatial context; constrained by the prior information	No	https://github.com/Teichlab/cellphonedb	Python	2020	[90]
CellChat	CCI	A tool to quantitatively infer and analyze intercellular communication networks from scRNA-seq data	Take multi-subunit protein complexes into consideration, as well as other important signaling cofactors	Cannot incorporate spatial context; constrained by the prior information	No	https://www.cellchat.org/	R	2021	[91]
NicheNet	CCI	A method to predict ligand–target links between interacting cells by combining their expression data with prior knowledge on signaling and gene regulatory networks	Incorporate both ligand–receptor interactions and intracellular signaling into prior model	Neglect multi-subunit protein complexes	No	https://github.com/saeyslab/nichenetr	R	2020	[92]
Giotto	CCI; SVG; clustering	A toolkit for characterizing cell-type distribution, spatially coherent gene expression patterns, and CCIs	Incorporate spatial context	Focus on unsupervised correlation-based analysis	Yes	https://github.com/RubD/Giotto	R	2021	[31]
Trendsceek	SVG	A method to identify significant gene expression gradients and hot spots in low-dimensional projections based on marked point processes	Incorporate both spatial and expression-level information	Analyze on normalized expression rather than count data	Yes	https://github.com/edsgard/trendsceek	R	2018	[81]
SpatialDE	SVG	A statistical test to identify genes with spatial patterns of expression variation from multiplexed imaging or spatial RNA-seq data	Relate tissue structure and cell type composition using the expression patterns of marker genes; computationally efficient	Analyze on normalized expression rather than count data	Yes	https://github.com/Teichlab/SpatialDE	Python	2018	[82]
SPARK	SVG	A method based on linear spatial models for identifying genes that display spatial expression patterns	Construct model based on count data directly; produce well calibrated *P* values for type I error control	Relying on pre-specified spatial kernels may limit its detection of genes not captured by them	Yes	https://github.com/xzhoulab/SPARK-Analysis/	R	2020	[83]
SpatialDE2	SVG	An integrated software framework which can unify the mapping of tissue zones and SVG detection	Deal with raw mRNA counts; offer superior computational speed	Require that the size of kernel and distance matrices scales with the square of the number of spatial locations	Yes	https://github.com/PMBio/SpatialDE			[84]
HRG	SVG	A method proposed to find the informative genes	Deal with both single-cell data and spatial data	Require comprehensively evaluation of performance on ST data	No	https://lifeome.net/software/hrg	R	2022	[85]
stLearn-PST	Trajectory analysis	A method to reconstruct the evolutionary trajectories based on transcriptome profiles and the spatial context of cells within a tissue	Incorporate spatial context	Analyze based on PAGA method	Yes	https://stlearn.readthedocs.io/	Python	2020	[48]
Monocle	Trajectory analysis	An unsupervised algorithm to increase the temporal resolution of transcriptome dynamics using scRNA-seq data collected at multiple time points	Infer more complex topologies	Cannot infer graph trajectory topology; time consuming	No	https://monocle-bio.sourceforge.net/	R	2014	[96]
PAGA	Trajectory analysis	An interpretable graph-like map of the rising data manifold to reconcile clustering with trajectory inference	Perform better on datasets with trees or more complex trajectories	Require prior information	No	https://github.com/theislab/paga	Python	2019	[97]
Slingshot	Trajectory analysis	A method for inferring cell lineages and pseudotime from single-cell gene expression data	Perform better on datasets containing more simple topologies; user-friendly	Cannot infer graph trajectory topology	No	https://github.com/kstreet13/slingshot	R	2018	[98]
SCORPIUS	Trajectory analysis	A trajectory inference method for inferring trajectories in a purely data-driven manner	Produce slightly more stable results	Can only infer linear trajectory topology	No	https://github.com/rcannood/SCORPIUS	R	2016	[99]
SIRV	RNA velocity	A method to derive cellular differentiation dynamics in a spatial context at the single-cell resolution	Incorporate spatial context	Cannot perform RNA velocity without spatial context	Yes	https://github.com/tabdelaal/SIRV	Python	2021	[103]
Velocyto	RNA velocity	A package for the analysis of expression dynamics in scRNA-seq data	Propose RNA velocity at the first time	Cannot incorporate spatial context; difficult to be applied to most transcription factors	No	https://velocyto.org	Python	2018	[104]
scVelo	RNA velocity	A likelihood-based dynamic model to generalize RNA velocity estimates for transient systems and systems with heterogeneous subpopulation kinetics	Infer RNA velocity in near-linear runtime	Cannot incorporate spatial context; difficult to be applied to most transcription factors	No	https://scvelo.org	Python	2020	[105]
Dynamo	RNA velocity	A computational framework to infer absolute RNA velocity, predict cell fates, extract underlying regulations, and predict optimal reprogramming paths and perturbation outcomes	Overcome the fundamental limitations of conventional splicing-based RNA velocity analyses	Cannot incorporate spatial context; require the steady-state assumption	No	https://github.com/aristoteleo/dynamo-release	Python	2022	[106]

*Note*: ST, spatial transcriptomics; scRNA-seq, single-cell RNA sequencing; RNA-seq, RNA sequencing; SVG, spatial variable gene; CCI, cell–cell interaction; 3D, three-dimensional; HRG, Highly Regional Genes.

## ST data exploration

ST brings new challenges to big data computing, visualization, and storage due to the generation of multiplexed and high-dimensional data that contain multicellular-, cellular-, or subcellular-level *in situ* gene expression information. For spatial barcode-based technologies, using Stereo-seq as an example, tens of terabytes of raw sequencing data can be generated from one tissue slice using ST technologies with high resolution and a large field of view; the results can include as many as tens of billions of capture units. For ISS-based and ISH-based technologies, the medium for spatial gene expression data is a series of images, which require image segmentation and recognition to extract the gene expression information and spatial locations. Generating a gene expression matrix, visualizing tens of billions of probes and storing, and accessing trillions of megabytes of data have propelled the use of big data in bioinformatics to a new level. Novel bioinformatics tools and databases are required to handle these new types of data.

### Data acquisition

**Figure 2:**
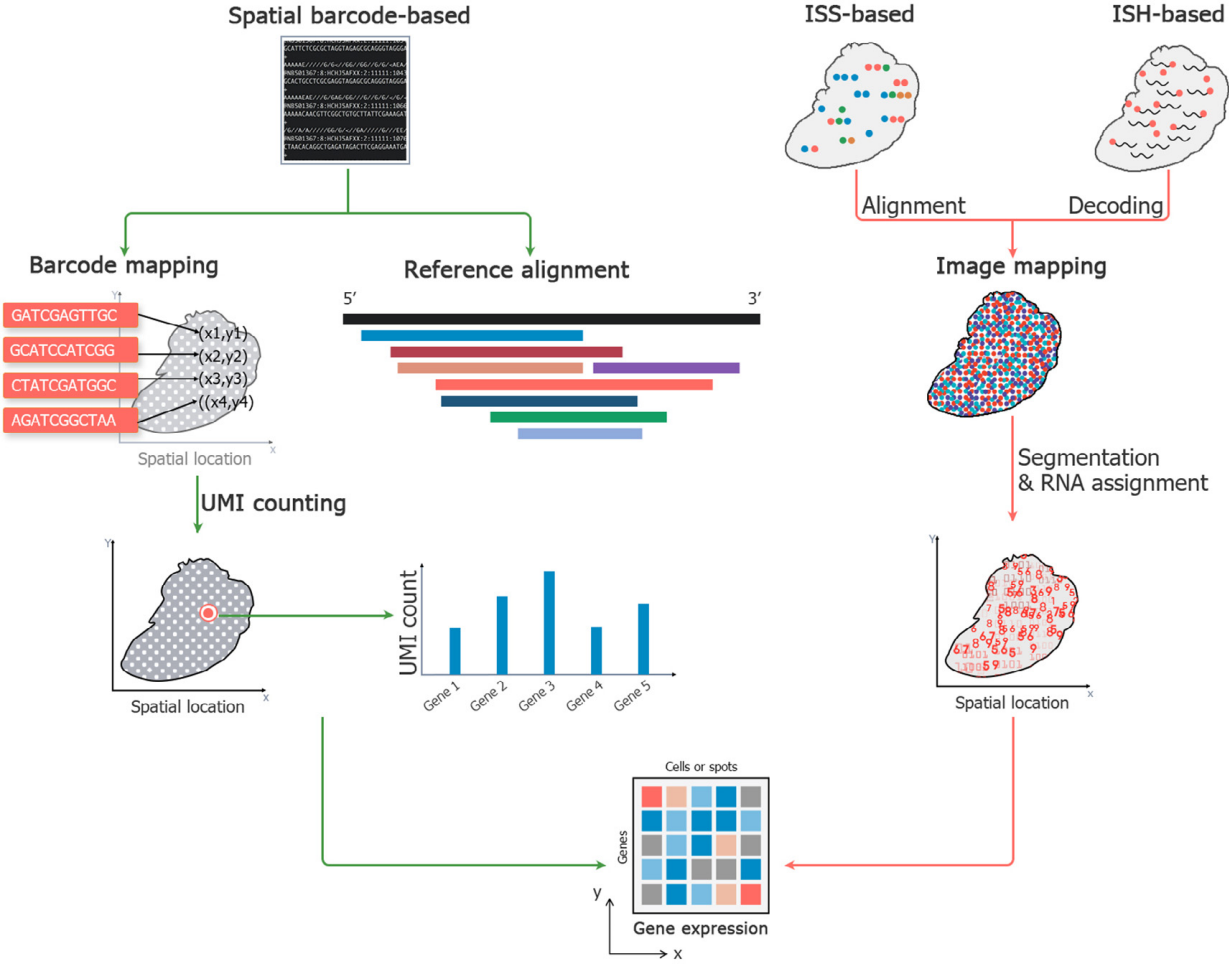
**Data computing to obtain gene expression information from raw sequencing or image data** The raw sequencing data generated from spatial barcode-based transcriptomics technologies contain two kinds of sequence information, barcodes and RNA sequences. The barcodes are mapped back to the spatial location, and corresponding RNA sequences are aligned with a genome reference. UMI counting is performed to count the number of aligned genes belonging to each cell or spot, and the gene expression profile is generated. The imaging data generated using ISS-based or ISH-based technologies can be transformed into images containing RNA signals by alignment or decoding. Image segmentation is used to isolate the RNA signals and each RNA is assigned to each spot or cell afterwards. Gene expression information with spatial locations is obtained. UMI, unique molecular identifier.

Typically, similar to single-cell RNA sequencing (scRNA-seq) data processing, obtaining the gene expression matrix from the raw sequencing data generated by barcode-based ST technologies is a standard analysis strategy that involves barcode mapping, alignment or assignment of reads, quality control, and unique molecular identifier (UMI) counting of RNA transcriptomes (Figure 2). Two classes of algorithms and tools have been developed to quantify gene expression. Full-alignment methods like Space Ranger (https://support.10xgenomics.com/spatial-gene-expression/software/pipelines/latest/what-is-space-ranger) use standard RNA sequencing (RNA-seq) aligners such as STAR [23] to align reads to a reference genome; this is a widely used standard solution for scRNA-seq or ST technologies. Pseudo/transcriptome alignment methods, including Alevin [24] and kallito|bustools [25], [26], [27], apply *k*-mer-based counting algorithms [27] or quasi-mapping [28] to boost the alignment efficiency by an order of magnitude, but the quantification accuracy is questionable [29]. Comprehensive benchmarks are needed to evaluate these methods under the ST context. Spatial barcode mapping of tens of billions of spatial locations is difficult and time-consuming, especially if sequencing errors and the collision rate are taken into consideration. The larger the number of spatial capture units is, the more likely it is that collisions will occur during the barcode mapping process. One way to solve this problem is to increase the length of the barcode sequence. Unfortunately, doing so also increases the error rate for the barcode sequence and further complicates the error correction procedure. Compared with the barcode mapping methods used in scRNA-seq, spatial barcoding is more complex and requires more efficient computational solutions.

Images generated by ISS-based and ISH-based technologies should be processed to obtain the cell-level matrix as well as the corresponding spatial location (Figure 2). Watershed algorithms and many deep learning methods can be applied to distinguish cell borders according to RNA density. Therefore, the identification of cell borders depends on the performance of the algorithms and the image quality, which suggests that the predicted cell boundaries may not be the actual physical boundaries, making it more difficult to assign each mRNA to the correct cell. Although data errors are inevitably retained, these images are eventually transformed into spatial expression matrices.

### Data visualization

An interactive and editable visualization interface is a practical solution for understanding and analyzing complex spatial data, as it turns pale granular data into easily understood and visually compelling information without the need for coding skills. However, the spatial gene expression interface has a high resolution, involves a large amount of data, and is cumbersome to operate. A more efficient visualization framework is therefore required to display tens of billions of data points and handle computationally intensive tasks.

With the rapid development of ST technologies, companies and teams involved in this area have put effort into developing interactive visualization and analysis tools. Desktop software, such as ST viewer [30] developed by SciLifeLab in Sweden and the 10X Genomics Loupe Browser (https://www.10xgenomics.com/products/loupe-browser), include a user-friendly real-time visualization interface for basic analysis and visualization. Loupe Brower also contains functionalities for more advanced clustering analysis, differential gene expression analysis, and spot–tissue image alignment. Giotto [31] is a web-based visualization tool that uses a Google Map-like algorithm to present and navigate ST data interactively. Its analysis module contains advanced real-time analysis functions such as exploring spatial patterns and cell-to-cell interactions. StereoMap (https://stereomap.cngb.org) is a web-based visualization interface designed by BGI Research to visualize subcellular resolved transcriptomic and tissue morphological data in a responsive and interactive manner. It employs a multi-resolution visualization system to efficiently navigate an extensive amount of data and provides utilities to select a subset of regions of interest for further advanced analysis. These visualization and analysis tools can visualize the arrangement of spatial locations or project the dimensionally-reduced data into 2D space. The gene expression value, cell cluster, and meta information of each cell or spot can be used to define the color and size of the scatter. However, these systems don’t support visualization of data features calculated by advanced analysis, such as that for the cell differentiation trajectory. In addition, these tools do not include three-dimensional (3D) visualization of multiple tissue sections due to the complexity. 3D visualization involves many steps, and it has a lot of problems that need to be settled. For example, tissue segmentation and registration, which is needed to achieve 3D visualization, are steps that can be improved with technological advances. This topic is explained in detail in the “3D reconstruction” section under the heading “Tissue-wide ST data interpretation”.

### Data storage and access

Storage challenges are encountered with three types of data: images, sequencing data, and their corresponding gene expression matrices. Images are commonly compressed and stored, which is a technically mature approach. Compared with whole-genome sequencing, the sequencing data volume for ST may be up to two orders of magnitude larger [12]. However, only a few existing sequence compression algorithms [32], [33] are tailored for transcriptomics data, and the location information for genes is incompatible with algorithms for data streams in FASTQ. Moreover, the gene expression information for adjacent locations has potential reference value for eliminating redundancy. Thus, customized algorithms are required to reduce the high cost of data storage. In terms of gene expression arrays, even though some tools have been developed for visualization and analysis as mentioned above, they are still unfit for prospective utilization with ST. Without a standard data format for gene expression arrays, various binary files with similar content would be created by different tools, which hinders the combination of tools and results in the waste of storage and calculation resources. To ensure high performance and a low cost of usage and updates, the data format needs to support a high input/output (I/O) speed, high compression ratio, and high scalability. Some application programming interfaces (APIs) are also required for specific usage scenarios, *e.g.*, real-time interactions for visualization. To date, a unified data format accompanied by a set of visualization and analysis tools is still unavailable.

Databases are necessary to collect, dispose of, integrate, and display ST data. SpatialDB [34] is reported to be the first manually curated database for spatially resolved transcriptomics techniques and datasets. It contains 24 datasets (305 sub-datasets) from five species (human, mouse, zebrafish, *Drosophila*, and *Caenorhabditis elegans*) generated using eight spatially resolved transcriptomics techniques. Besides SpatialDB, only a few cellular or molecular atlases for specific tissues are available for data acquisition [35], [36], [37], [38]. In contrast, databases developed for single-cell datasets are more comprehensive and each has different limitations. PanglaoDB [39], single-cell studies database [40], and scRNASeqDB [41] utilize single-cell datasets with the corresponding experiment information and display the gene expression. The EMBL-EBI Single Cell Expression Atlas (https://www.ebi.ac.uk/gxa/sc/home) and Single Cell Portal (https://portals.broadinstitute.org/single_cell) provide data files after reprocessing using predefined processing pipelines. Cell BLAST [42] and CellAtlasSearch [43] apply trained cell-type classification models using the collected data to predict the cell type of a query cell. The recently released hECA [44] database assembles the collected data into a unified data repository for overall screening, computing, and data mining. Based on its collected single-cell datasets, the newly published DISCO [45] database constructs one global atlas and 27 sub-atlases for different tissues, diseases, and cell types.

Databases such as those listed above, which were created for data collection, sorting, reprocessing, integration, and providing retrieval and query services, should be established for ST technologies as well. Compared to single-cell data, cells or spots with spatial locations from the same tissues pose the problem of integration at the tissue level. As has been shown, it takes 190 s to screen 210,000 cells from 1,093,299 cells in hECA using a logic expression [44]. Data screening of all studies, meaning all tissues and all cells have been screened within an acceptable time frame, is the first challenge for big data storage. Fast calculation for data reprocessing and data similarity is the second challenge for big data computing. These two challenges require database creators to focus on the comprehensive design of databases.

## ST data quality control and preprocessing

**Figure 3:**
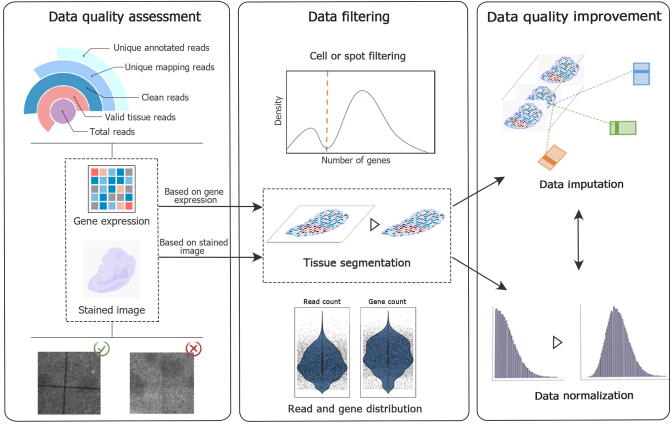
**Data preprocessing for ST data quality control** Data quality assessment is the first step to evaluate and determine whether the data are ready for the next analysis. Image data with high resolution and expression data with enough supported reads, including valid reads mapped to the tissue-covered region (valid tissue reads), clean reads, unique mapping reads, and the total gene number, are checked. Then, the data after tissue segmentation, which contain cells/bins and genes, is further screened to eliminate low-expression cells/spots and genes. Finally, data normalization and data imputation are applied to improve the data quality.

Due to the limitations of the current experimental methods, the number of genes effectively captured by barcode-based ST technologies is substantially lower than that for scRNA-seq. Specifically, the gene expression value extracted with sequencing technology is lower than the real expression level. Moreover, some genes known to be present in the sample may be missing entirely [46]. In addition, gene diffusion caused by the experiment and the inner batch effect is a common problem in ST data generated by barcode-based ST technologies. The quality of images generated by all ST technologies is highly dependent on the imaging system; therefore, images should be assessed before used for data collection. In summary, it is necessary to perform data quality assessment, data filtering, normalization, and data imputation to improve the data quality for subsequent analysis (Figure 3).

### Data quality assessment

Data quality is influenced by the experiment workflow, manual operator, sequenator, and imaging system. All of the data are mainly generated by the sequencer or imaging system and can be divided into two types: sequencing data and images. Sequencing data are processed to determine the gene expression, similar to the common RNA-seq data or scRNA-seq data, with the slight difference being that ST data contain spatial location information. Data quality assessment of sequencing data and data generated during subsequent processing are mainly focused on four indicators: (1) the valid reads or UMIs or RNAs that can be mapped to the tissue-covered region, (2) the amount of clean reads without an adapter and barcode sequence, (3) the unique mapping reads that can be mapped back to the unique locations of the reference genome, and (4) the amount of unique reads that can be successfully annotated.

Images generated by the imaging system can be divided into two types according to their usage: the fluorescence imaging data used by ISH-based and ISS-based technologies that contain molecular signals, and the stained images that present the details and contours of the tissue slice. The image quality is highly dependent on the resolution and stability of the microscope and its manual operation. The strategy used for the microscope will first generate several sub-images and subsequently combine them to obtain the full image. Reference coordinates are pre-generated by ST technologies and image stitching is an essential step to obtain a complete and correct view of the tissue slice. The Visium platform generates fiducial spots of each area in Visium slides to determine the location of the captured region and assist in image stitching. The Stereo platform uses the track line built into the chip for the same purpose. Therefore, the image clarity and resolution of the focal plane of sub-images are mainly evaluated for image quality assessment.

### Data filtering

It is necessary to determine which spots are covered by tissues and contain real transcripts for the data generated by barcode-based ST technologies. In ST experiments, the transcripts may become diffused during permeabilization and subsequent biochemical reactions, finally spreading to areas not initially covered by tissues. Some arbitrary screening can be performed to filter these areas, *e.g.*, by screening the number of UMIs to filter out spots with a low capture rate. However, such global filtering does not take into consideration the prior tissue shape. On the contrary, a more reasonable approach is to segment the tissue area according to the stained image. Space Ranger, which is designed to process 10X Visium spatial gene expression data, trains a statistical classifier to separate fiducial spot patterns covered by tissue from the background area. TissueCut (https://github.com/BGIResearch/TissueCut) developed for Stereo-seq technology also provides a deep neural network-based method for cutting out the tissue area, either from registered stained images or directly from the gene expression profiles. However, the unique appearance of each tissue sample and the variable scale, size, and quality of the stained image make the problem challenging to solve. Therefore, when automatic organization segmentation algorithms cannot provide an optimal solution, manual correction may be necessary. The gene expression information generated by ISH-based and ISS-based technologies may also require tissue segmentation when noise exists outside the tissue region associated with the probe binding.

The gene expression metrics are measured on the spot by most barcode-based ST technologies. For subcellular resolution data, data binning is performed to combine the genes in multiple spots or bins to achieve gene expression at the single-cell level. For low-resolution data, the spot is the minimum unit for measuring gene expression. The spatial gene expression should undergo some preprocessing operations before subsequent data correction and analysis. Low-quality cells or spots should be filtered according to the quality control metrics, such as the number of genes contained in each cell or spot (low-quality cells or empty droplets contain very few genes) and the percentage of mitochondrial genes (low-quality or dying cells often exhibit extensive mitochondrial contamination) [47]. Genes with few cells or spots detected (usually less than three cells) will also be screened. However, the filtering threshold needs to be adjusted based on the objectives of the study.

### Data normalization

The purpose of normalization is to counteract technical noise or bias in sequencing depth. Spatial morphology and gene expression measuring, combined in stLearn [48], is the first method that uses neighborhood information (spatial location) and morphological distance to normalize gene expression data. However, evaluation metrics to measure its performance are lacking.

Data normalization tools that are currently widely used in research are customized for scRNA-seq data and can be divided into two groups according to related principles. The first type of normalization method is log-normalization. A size factor representing the relative deviation of each cell is estimated to eliminate bias and generate the normalized gene expression. Scran [49] uses the size factor to deal with the dropout and zero count of scRNA-seq data. BASiCS [50] infers cell-specific size factors based on spike-ins to distinguish technical noise from biological cell-to-cell variability. However, high-abundance genes may not be effectively normalized using this normalization method [51]. The other approach is based on a probability model and represents a relatively new and complex strategy. It simulates the counting of small molecules by fitting distributions and uses model fitting residuals as a standardized quantification of gene expression. Some methods such as ZINB-WaVE [52], scVI [53], and SCTransform [54] (wrapped in Seurat) were developed based on the aforementioned idea. SCTransform, recommended by Seurat, fits a regularized negative binomial model to raw count data. The residuals of this model can be used as normalized and variance-stable values.

Due to the widespread confusion in single-cell experiments and the lack of uniform optimal standardization among datasets, sometimes a single method may not be enough to normalize a dataset. It is a good strategy to use multiple statistical indicators to guide the selection of appropriate methods for a given dataset [55]. Besides, the distribution and sparsity of ST data differ from those of single-cell data, and the validity of single-cell data normalization algorithms applied to ST data needs to be verified. Spatial location and imaging information can also effectively assist with the smooth normalization of spatial expression and reduce the real differential expression. Furthermore, a positional batch effect may be present, that is, genes expressed in different locations in the same tissue can be significantly different in some cases. However, existing normalization methods do not address these issues. Taking these data characteristics and issues into account, the requirements for new algorithms for normalization should include the ability to obtain more realistic spatial gene expression, and to differentiate ST data from single-cell data.

### Data imputation

Imputation aims to accurately predict the expression of unmeasured genes and effectively identify cell populations with low gene detection sensitivity [11]. Data imputation is not always necessary for ST data analysis. Usually, it is required for data generated by barcode-based ST technologies because the data are relatively sparse compared to the data generated by other ST technologies. Some techniques apply data imputation by integrating ST data and single-cell data. Linked inference of genomic experimental relationships (LIGER) [56] and Seurat [57] are joint embedding-based methods, which combine dimensionality reduction for single-cell data and ST data and then impute the unmeasured genes of ST data based on the connection between cells in the two datasets. gimVI [58], a deep generative model, aims to solve the problem with imputing missing genes in ST data based on single-cell data from the same biological tissue. This model can use all the genes expressed in single-cell data for imputation, but which genes contribute to the imputation task is not clear in the prediction task. SpaGE [59] only uses the genes shared by the spatial and single-cell data to perform linear joint embedding and then predicts spatial gene expression through the k-nearest neighbor (k-NN) method, which can predict the whole transcriptome expression in the corresponding spatial configuration. Instead of only using genes shared with ST data, stPlus [60] builds an autoencoder to leverage overall information from reference scRNA-seq data. Additionally, stPlus predicts the gene expression in ST via a weighted k-NN approach by learning cell embeddings. The methods described use scRNA-seq data as the reference to predict ST data; however, they do not fully utilize the association between genes and the gene features that appear only in ST data. Furthermore, these methods neglect to take advantage of spatial and histology information, which have critical complementary functions for gene imputation. It is important to note that the majority of imputation methods do not improve performance in downstream analysis compared to no imputation, especially for clustering and trajectory analysis according to evaluations at the single-cell level [61]. Therefore, imputation should be used with caution. To improve the value of imputation, a comprehensive evaluation of these methods applied to ST data is urgently needed.

## Single cell- and tissue-level annotations in ST data

**Figure 4:**
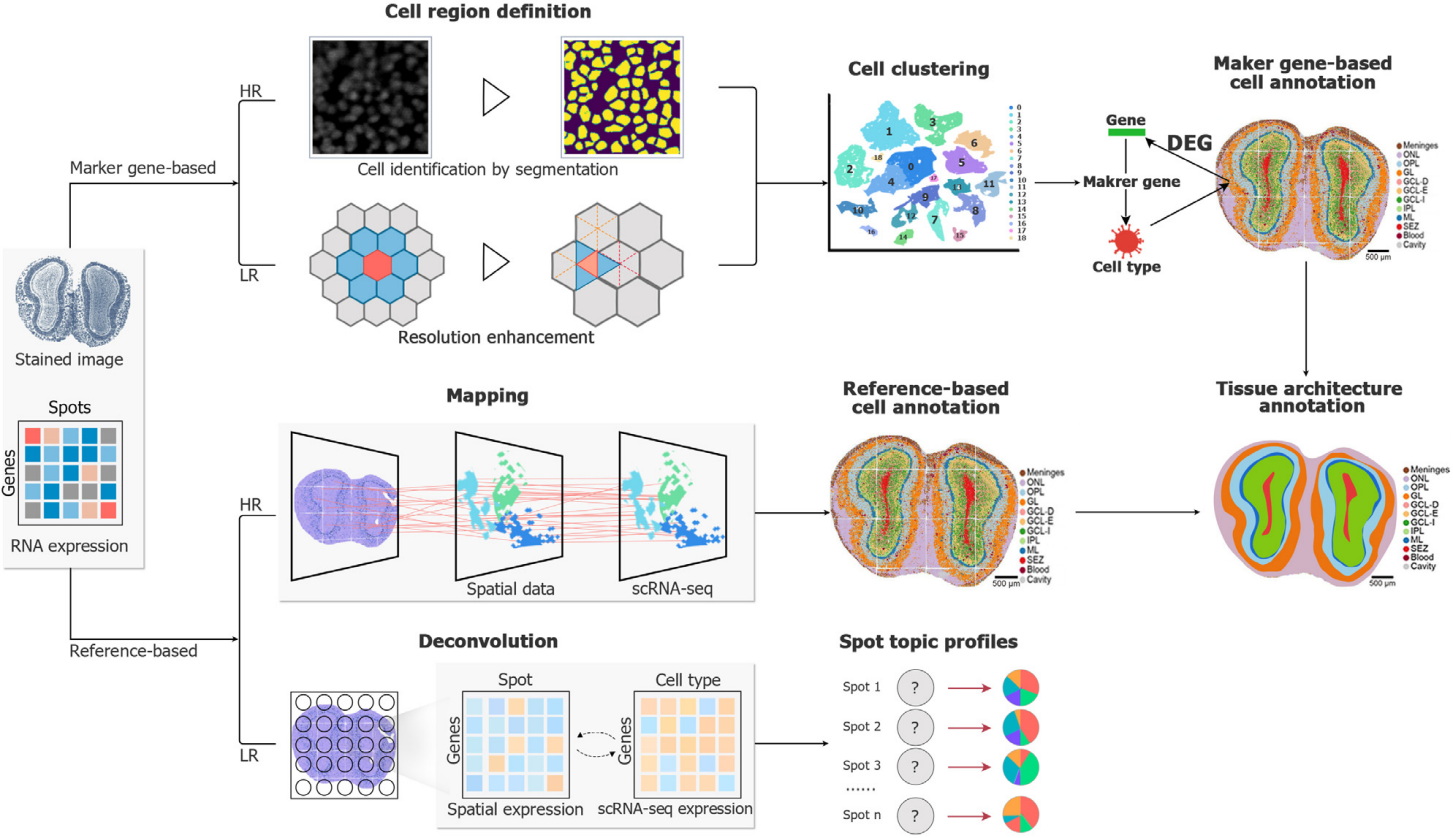
**Cell- and tissue-level annotationsfor high- or low-resolution data** There are mainly two solutions for cell annotation of the ST data: marker gene-based annotation and reference-based annotation. Marker gene-based annotation includes cell region definition, cell clustering, and cell cluster annotation. With this solution, the ST data with subcellular resolution are used to perform cell identification. This is implemented by cell segmentation based on stained images to obtain the cell boundaries and to identify the areas with relatively concentrated gene expression signals. For low-resolution data, computational approaches can be applied to enhance the resolution. Clustering and annotation based on marker genes are then applied to provide more comprehensive definitions of the cells. Reference-based annotation integrates scRNA-seq data to annotate cell types or cell-type composition by deconvolution for low-resolution ST data or by direct mapping for high-resolution ST data. Tissue architecture annotation can be further applied to obtain the tissue architecture based on cell clustering information. scRNA-seq, single-cell RNA sequencing; DEG, differentially expressed gene; HR, high resolution. LR, low resolution.

One of the key advantages of ST techniques over scRNA-seq is the ability to associate spatial information to gene expression patterns. The introduction of spatial information facilitates single cell-level annotation and the study of tissue architecture and function. There are mainly two solutions for cell annotation of the ST data: marker gene-based annotation and reference-based annotation (Figure 4). The marker gene-based annotation involves multiple steps including cell identification and reads assignment, followed by clustering and then marker gene-based annotation. The reference-based annotation makes use of databases or references generated by other techniques, specifically scRNA-seq, to guide automatic cell annotation. Tissue structure annotation is further applied to identify biologically heterogeneous regions and may support the study of biological and disease development.

### Cell-level marker gene-based annotation

Cell annotation, cell typing, or cell-type mapping provides the foundation for subsequent in-depth analysis, including the study of tissue function and cell–cell interactions (CCIs) in the microenvironment. This perspective considers the conventional cell annotation, which is a multi-step procedure: cell definition by allocating mRNA reads and optional cell segmentation, clustering analysis on gene expression features, and finally cell-type annotation by identifying marker genes that match prior biological knowledge.

There are different ways to define a cell region in ST data. For low-resolution ST techniques such as 10X Visium, mRNA reads are captured within patterned spots. Therefore, the minimum processing unit is a spot, which is considered a pseudo cell, even though each spot contains multiple cells. In order to cope with low resolution, resolution enhancement methods have been proposed. BayesSpace [62] splits the Visium spots into subspots and estimates the gene expression level at each subspot by leveraging the spatial neighborhood structure. XFuse [63] uses a generative network to produce a high-resolution expression map by integrating spatial gene profiles with histology images. As a preprocessing step, resolution enhancement can reveal cell composition in detail as well as the tissue’s finer anatomical structures. On the contrary, for high-resolution ST techniques, in order to properly assign mRNA reads to each cell region, one approach is to “bin” the reads with a proper radius. This can be done by simply considering the region within a square or circular area as a cell, or by estimating the cell span with a probabilistic model that estimates the gene expression distribution [64]. Although spatial binning divides cell regions without the need of additional information, the grid or regular patterns of the bins only give an approximation of the cell shape, while the probabilistic model-based estimation produces irregular cell boundaries, which significantly affects downstream analysis, including interpretations of CCIs. For ST technologies that come with fluorescence imaging, cell segmentation methods can be applied to obtain cell boundaries. Qian et al. [65] used traditional watershed segmentation, and Littman et al. [66] incorporated Cellpose [67] to detect the cell nuclei in 4,6-diamino-2-phenyl indole (DAPI)-stained images. HDST [8] performs nuclei segmentation on hematoxylin and eosin (H&E) images by combining Ilastik [68] and CellProfiler [69]. Segmentation algorithms have been widely investigated in the area of histological and fluorescence images, and hence can be easily adjusted to meet the needs of ST data. However, the accurate delineation of cell nucleus boundaries remains challenging due to problems such as touching or overlapping cells and defocusing aberrations. Most importantly, the nucleus segmentation is just an estimation of the whole-cell region, and it is better to correct the assigned mRNA reads to reveal the true nature of the cell.

Once the genes are assigned to corresponding cells, clustering analysis is then performed to provide a general overview of the morphological structure of the tissue. The clustering workflows proposed for scRNA-seq analysis, such as in Seurat V3 [47] and SCANPY [70], are commonly used in ST analysis, where traditional Leiden or Louvain clustering is often applied on a feature set generated by performing dimensionality reduction with methods such as principal component analysis (PCA), t-distributed stochastic neighbor embedding (t-SNE), and uniform manifold approximation and projection (UMAP) on gene expression data. However, these approaches only employ gene expression information and ignore the strength of ST datasets. Recently, several spatial clustering and feature extraction algorithms were developed to combine spatial distance information or tissue morphological information with gene expression profiles. For example, BayesSpace [62] implements a fully Bayesian model with a Markov random field before encouraging neighboring spots to join the same cluster. MUSE [71] constructs a multi-view autoencoder to combine multi-modal information to identify high-resolution latent subpopulations. It can discover tissue subpopulations missed by either modality as well as compensate for modality-specific noise. BANKSY [72] combines cell expression with the weighted average of the expression of neighboring cells within its microenvironment as augmented features for spatial clustering. SpaGCN [73] and structural elements detection and reconstruction (SEDR) [74] use deep autoencoder networks to integrate the spatial distance and gene expression to generate a low-dimension representation for clustering. The embedding generated by a deep autoencoder model can then be used for subsequent tasks. Although most methods present reasonable results, prior biological knowledge and, if possible, proper ground truths are needed to evaluate the clustering results. Methods that require the tuning of hyper-parameters suffer from subjective assessment, where a perturbation in parameters could result in distinct spatial patterns. Methods that incorporate spatial information sometimes introduce excessive spatial influence, producing spatially lumpy clusters, which deviate from the true biological structures. With regard to the importance of clustering, the selection of a proper methodology should be determined with caution.

Cells of the same cluster have similar gene expression levels, and thus are considered to be the same cell type. The topmost differentially expressed genes (DEGs) are usually used to characterize the biological functions of the cells, which is the basis of naming a cell type by biologists. Therefore, one can manually associate cell types to clusters by matching the marker genes to existing knowledge. However, as straightforward as it seems, manual annotation is laborious and prone to error. Marker gene detection is not always exclusive, meaning a gene which defines a cell type can exist in more than one cluster, therefore leading to similar rankings between clusters. Sometimes a cell type is determined by the coexistence of several genes, but not all of them are ranked at the top in any clusters, which complicates the decision. Moreover, conflicts occur in prior knowledge, as the annotation standards vary among studies. Therefore, it is important that researchers have relevant background knowledge and experience in order to perform these analyses with good judgement.

### Cell-level reference-based annotation

Thanks to the extensive research in scRNA-seq, numerous cell-type databases have been introduced for various types of species and tissues, which make annotating ST data with existing references possible. Based on resolution differences among various ST techniques, cell annotations with references can be further divided into two categories: deconvolution and direct mapping.

Due to technical limitations, ST technologies such as 10X Visium, which has spots with a minimum diameter of 55 μm, are not able to achieve single-cell resolution. In order to properly annotate cell identities, deconvolution algorithms have been proposed to estimate the composition of cells within each physical spot by transferring cell-type signatures defined by scRNA-seq. Robust cell-type decomposition (RCTD) [75] fits each pixel as a linear combination of individual cell types and estimates the cell-type proportion by fitting a statistical model to the assumed Poisson-distributed gene expression levels. SPOTlight [76] focuses on a seeded non-negative matrix factorization regression to deconvolute ST spots using predefined cell-type marker genes. SpatialDWLS [77] uses an enrichment analysis to find potential cell types at each location, and then a dampened weighted least squares method is used to infer the cell-type composition. Cell2location [63] first estimates the scRNA-seq reference cell-type signatures by a negative binomial regression model, and then the hierarchical Bayesian model is used to decompose the mRNA counts in the multi-cell ST data to obtain accurate cell-type subpopulations. Although a deconvolution strategy can predict the cell-type composition, each spot is still a mixture of several different cells, thus making a high resolution-ready ST technology exceedingly compelling.

For ST techniques that achieve cellular or subcellular resolution, cell annotation is performed by first assigning gene expression to each predicted cell location, followed by direct scRNA-seq cell-type mapping. pciSeq [65] first detects the cell nuclei in DAPI-stained images using traditional watershed segmentation, then uses Bayesian modeling to estimate the probability for allocating each read to each cell, and eventually assign each cell to its proper cell class. Baysor [78] combines the spatial density, position, and gene identity to estimate neighborhood composition vectors for each molecule, and utilizes the Markov random field to assign neighboring molecules to the same cell, which can be annotated according to scRNA-seq data. Joint cell segmentation and cell-type annotation (JSTA) [66] first performs watershed-based segmentation on DAPI-stained images to extract cell nuclei and their corresponding RNA signals in MERFISH data. A deep neural network is then trained on scRNA-seq data to assign cell types to each segmented cell. The cell classification result is iteratively updated by reassigning pixels to new labels according to the estimated probabilities. Spot-based spatial cell-type analysis by multidimensional mRNA density estimation (SSAM) [64] first creates a gene expression vector field by calculating and stacking the mRNA intensity distribution of each gene in fluorescence *in situ* hybridization (FISH). A cell-type map is then generated by labeling each pixel in the image according to the computed gene expression signatures. The aforementioned methods are applied on ISH- or ISS-based technologies, while CellTrek [79] is applied on barcode-based technologies. CellTrek combines ST and scRNA-seq data into a shared latent space and trains a random forest model to map spatial coordinates to single cells which are close in distance to their corresponding co-embedded ST counterparts. However, CellTrek maps cells to their most similar spots based on a sparse graph, which requires ST spots with relatively high cell purities.

With the help of the available scRNA-seq annotation database, cell-type knowledge can be transferred to ST data by matching the closest features defined and extracted by the proposed methods to existing reference profiles. The annotations of cell types have high confidence as the scRNA-seq database has been proven to be credible. However, it is difficult to find new cell types that are not present in the database. Moreover, since the gene expression information obtained with scRNA-seq has rather distinct characteristics compared with that of the current ST data, normalization and correction of the transferred data are required.

### Tissue architecture annotation

The aforementioned methods are mainly focused on predicting or decomposing cell types; however, not all of them seek accurate tissue composition. Algorithms such as SpaGCN [73] and BANKSY [72] have the ability to perform spatial domain segmentation, providing insights into a tissue’s anatomical structure, while other methods can distinguish the spatial composition of cells without determining the actual biological outlines. The recently proposed RESEPT [80] framework aims to accurately segment the tissue architecture. Gene expression is first encoded to a three-channel representation with a graph autoencoder, and then a convolutional neural network-based image segmentation model is used to obtain the spatial functional regions. The pathway enrichment analysis is further used to confirm the biological validity of the segmented tissues. Tissue architecture is the key element necessary to understand the function of organs; therefore, it is crucial to identify the biologically heterogeneous regions within a tissue. The anatomical information contained in images is essential for tissue architecture annotation tasks. With accurate functional zonation, knowledge such as the pathogenesis of human diseases as well as development-related functional evolution can be further attained.

## Tissue-wide ST data interpretation

ST technologies allow researchers to identify the characteristics of cells at the single-cell level and study the spatial associations of cells and genes in multi-dimensional spaces in combination with spatial locations and tissue images. This includes detection of spatial variable genes (SVGs) in spatial tissue domains, uncovering the CCIs and gene–gene interactions involved in cellular communications and collaboration, inferring the dynamics of cell-state transitions and cell fate through trajectory and RNA velocity analyses, and discovering new insights in the 3D space. Tissue serial slices and time-series slices place higher demands on data interpretation in terms of space and time dimensions.

### SVGs

Identification of SVGs that display spatial expression patterns is critical for characterizing the ST landscape in tissues. Trendsceek [81] applies marked point processes to reveal significant gene expression gradients and hot spots in low-dimensional projections. SpatialDE [82] and SPARK [83] identify SVGs based on non-parametric Gaussian process (GP) regression, with SPARK additionally offering a count-based likelihood and a more powerful statistical test. Trendsceek and SpatialDE use normalized expression values for analysis, which can be suboptimal compared to count data used by SPARK [83]. SpatialDE2 [84] is an integrated software framework released recently, which can unify the mapping of tissue zones and SVG detection. Extended features of SpatialDE are that it can manage raw mRNA counts and offer superior computational speed over previous methods [84]. These described methods treat the spatial distribution of gene expression as image patterns, and the gene analysis focuses more on comprehensive information rather than neighborhood expression patterns. The biological significance of the findings using these methods needs to be further examined. Identifying Highly Regional Genes (HRG) [85] is a newly published method designed to detect informative genes with expression levels regionally distributed in adjacent cells. The cell–cell network is constructed by the cell–cell distances in low-dimensional space for scRNA-seq datasets or constructed by spatial distance for ST datasets.

SVGs are similar to “highly variable genes” proposed in single-cell datasets. The major difference is that the detection of SVGs takes spatial information into consideration. Thus, the informative genes are detected within the continuous spatial tissue regions, which makes the variable genes more reliable and significant. SVGs are detected to study the spatial expression patterns in spatial tissue zones, while DEGs are calculated to define the specificity of a certain cluster compared to other clusters. DEGs can be easily identified by performing a statistical testing, including the *t*-test and Wilcoxon rank-sum test, which are wrapped in several tools, such as Seurat [57] and SCANPY [70]. Combining the studies of SVGs and DEGs may lead to a more comprehensive interpretation of the microenvironment of spatial tissues and the independence and cooperation of cell groups.

### CCIs

**Figure 5:**
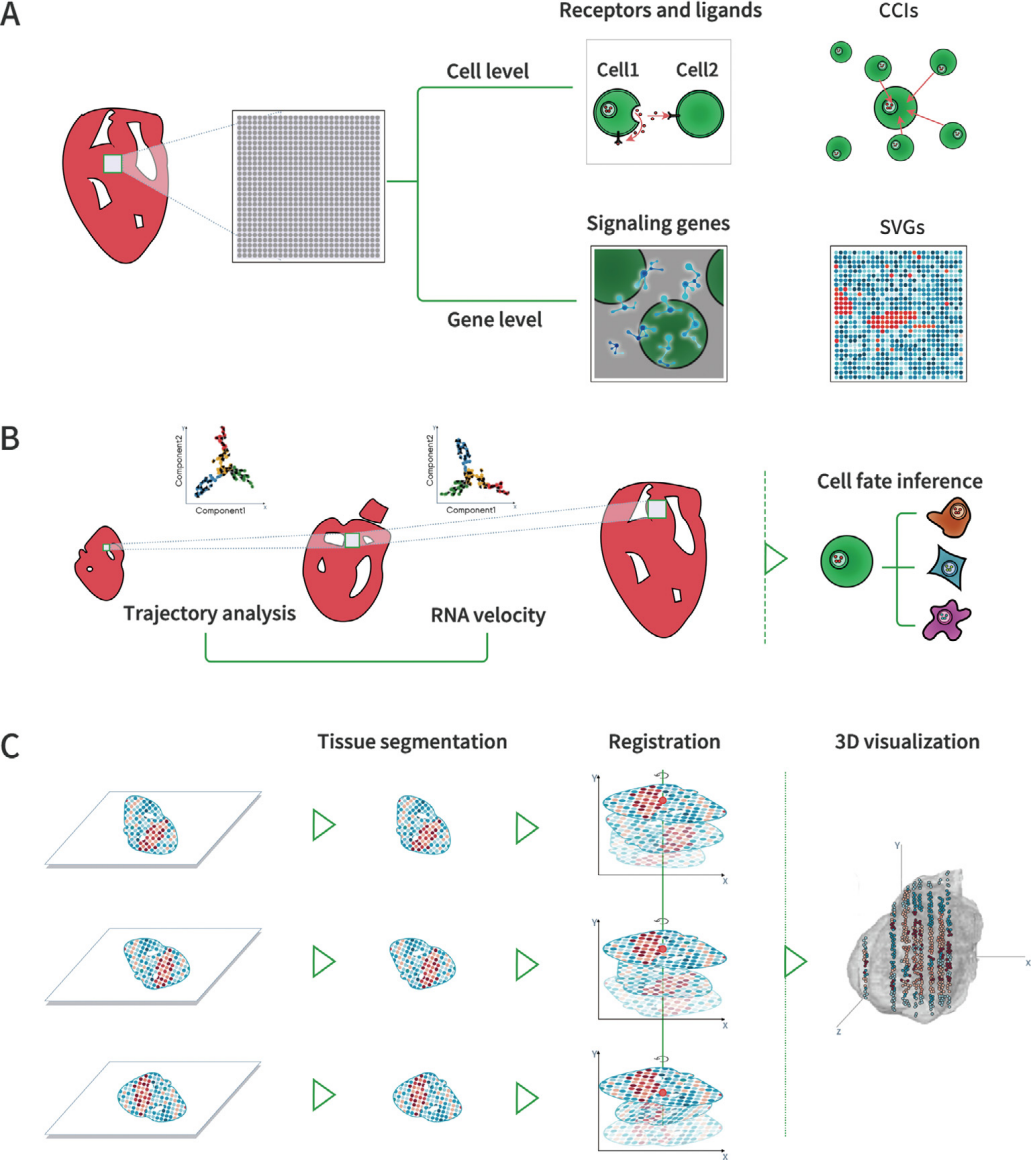
**Tissue-wide data interpretation includes single or multiple slices in two- or three-dimensional space** **A.** Cell communication and gene behavior can be predicted based on spatial gene expression. The receptors and ligands can be identified to further predict cell communication, and key signaling genes and SVGs can be identified as another aspect to study cell behaviors. **B.** Trajectory analysis and RNA velocity are used to predict the cell state dynamics and infer the cell fate particularly in time-series slices to study cell development. **C.** 3D reconstruction can be divided into three steps, namely tissue segmentation, registration, and 3D visualization. A 3D view of the tissue makes it possible to perform the aforementioned data mining from 2D to 3D data. 2D, two-dimensional.

ST technology provides a unique opportunity to understand CCIs, the signals of genes that mediate cellular characteristics across biological tissue and cellular organization (Figure 5A). The physical distance between cells is considered an essential attribute in the study of CCIs since it has the potential to illustrate the roles of cells during communication and interactions [86]. Most computational strategies are focused on ligand–receptor pairs and their designs are based on the cell–cell distance. Ligand–receptor pairs are known to produce signaling events that eventually lead to CCIs in the microenvironment and tissue structure [87]. Some approaches directly map single-cell data to ST data to obtain the spatial position of ligands and receptors [20], [88]. Other sophisticated tools designed for ST assign scores by calculating the interactions between cells and their neighbors, while also taking the cell-type into account. Giotto [31] ranks ligand–receptor pairs based on the cell–cell communication score to identify interactions between adjacent cells. The graph convolutional neural network approach for genes (GCNG) [89] builds graph convolutional networks to propose novel pairs of cells with extracellular interactions. Furthermore, expression permutation-based tools, such as CellPhoneDB [90] and CellChat [91], can infer CCIs directly at the single-cell expression level. These two methods take multi-subunit protein complexes into consideration and address the common limitation of many other methods by doing so. CellChat even incorporates signaling cofactors, including soluble agonists, antagonists, and stimulatory and inhibitory membrane-bound co-receptors. NicheNet [92] predicts ligand–target links between interacting cells by combining their expression data with more comprehensive prior knowledge, which incorporates ligand–receptor interactions and intracellular signaling. However, NicheNet neglects multi-subunit protein complexes and signaling cofactors. Although various computational approaches have emerged, it remains unclear which metric best captures the underlying CCIs. Therefore, different methods should be tested to evaluate the value of resulting data for each method [87].

Spatial and histological information is undoubtedly essential for CCIs. On one hand, cells in close proximity are more likely to interact than cells that are farther away from each other. On the other hand, the tissue region boundaries extracted from histological images are also key locations where important CCIs occur. Omics data from other dimensions, including proteomics data and glycomics data, can also be integrated methodically to improve the prediction of CCIs and signaling genes. It is important to note that the presence of ligand–receptor pairs inferred from transcriptomic data may be inconsistent with their actual presence in proteomic data. Glycosylation has effects on protein interactions, especially in ligand–receptor binding and can alter receptor affinity [87]. With the maturity of 3D ST reconstruction techniques, future methods should simultaneously analyze multiple consecutive sections of the same tissue to discover CCIs in 3D space. Furthermore, time-series slices require methods to elucidate important changes in CCIs during dynamic processes such as cellular differentiation and immune responses. Multi-omics, multi-dimensional, and multi-time-series data not only provide opportunities for novel discoveries but also pose a challenge for developing more comprehensive CCI and signaling gene mining methods.

### Cell fate inference

The cell fate decision refers to the future developmental fate of a cell, which is restricted and differentiated in a specific direction before a recognizable morphological change occurs. The application of trajectory and RNA velocity analyses provide essential insights into cell fate decision processes (Figure 5B).

Trajectory analysis aims to reconstruct the structure and dynamics of cell-state transitions by exploring asynchronous cellular behaviors, stochastic and regulated cell-to-cell variation, or intrinsic heterogeneities (including lineage diversification, lineage convergence, and re-specification of cell identity by cellular reprogramming) [93]. The phase stretch transform (PST) algorithm in stLearn reconstructs evolutionary trajectories based on the transcriptome profile and the spatial context of cells within a tissue [48]. It uses partition-based graph abstraction (PAGA) to identify connections within clusters and compute the pseudotime via diffusion pseudotime (DPT). Trajectory analysis methods applied in most ST studies are designed for single-cell analysis [94] and have been comprehensively reviewed and benchmarked by Saelens and his colleagues [95]. Among them, Monocle first places cells in a low-dimensional space and then predicts developmental trajectories by building a minimum spanning tree [96]. PAGA [97] generates graph-like maps of cells to preserve both continuous cell transitions and discrete cell types. Slingshot [98] takes advantage of both techniques to identify multiple branching lineages. SCORPIUS [99] infers trajectories in a purely data-driven manner without prior information about the dynamic process. These methods vary in their ability to detect different trajectory topologies: linear, tree (such as bifurcation and multifurcation), graph with cycles, and disconnected graphs [100]. In most cases, users know little about the expected trajectory of the dataset, but these methods preset the trajectory topologies in advance. According to benchmarking, there is no single best approach for all types of datasets [101]. Therefore, when users have sufficient knowledge about the dataset, an algorithm suitable for the corresponding trajectory topology of the dataset should be selected to complete the task.

RNA velocity analysis can predict the future expression status of cells to study the cell state dynamics; construct cell differentiation trajectories; estimate the rate of transcription, splicing, and degradation; and classify different cell dynamics mechanisms [102]. Spike-in RNA variants (SIRV) [103] is a recently published software that integrates ST and single-cell data to calculate the RNA velocity for each cell and derives the flow field by averaging the dynamics of neighboring cells. Without spatial information, some algorithms can be used to calculate RNA velocity at the single-cell level. Velocyto [104] is the first method to infer RNA velocity of single-cell transcriptomic data based on a steady-state model. It uses the disproportion of pre-mRNA compared to mRNA to estimate the expected mature mRNA profile of the corresponding future cell. scVelo [105] constructs a system of differential equations based on a likelihood-based dynamical model. It takes transcription as well as mRNA processing, degradation, and latent time into consideration to fit the mature mRNA and pre-mRNA levels. A steady-state model assumes the presence of steady states and a common splicing rate across genes while a likelihood-based dynamical model maintains weaker assumptions of constant gene-specific reaction rates of transcription, splicing, and degradation which are inferred by expectation–maximization (EM) framework. However, these assumptions may not be consistent with reality and these methods suffer inherent noisiness due to the limited read counts of pre-mRNA and the dependency on the selected genes [100]. These conventional splicing-based RNA velocity analyses are difficult to apply to most transcription factors, which are typically expressed at low levels and are related to genes with no polyA/T-enriched intron regions. Dynamo [106], a recently published method, integrates metabolic-labeling information for pre-mRNA age profiling to improve the RNA velocity estimation. It overcomes the fundamental limitations of conventional splicing-based RNA velocity analyses but still largely depends on the steady-state assumption.

Much of the work on spatial datasets relies on existing tools that allow trajectory and RNA velocity analyses without spatial information. The combination of spatial mapping and cell transition inference can provide probabilistic values for differentiation directions based on cellular proximity in spatial tissues. Thus, cell development trajectories, key cell subsets, and genes involved in differentiation can be revealed more accurately. Furthermore, it may unveil the transcriptional changes and mechanisms of anatomically restricted cell populations and provide novel insights into the molecular programs within developing tissues [100]. Spatial multi-omics data involved in trajectory and RNA velocity analyses can provide new insights into intracellular processes, especially the causes of lineage differentiation results. Time-series data at the single-cell level are directly integrated by these methods, but for ST data, methods to incorporate the relative independence and organic combination of tissue information and development time should be considered for future algorithms. In addition, inferring and visualizing cell state dynamics and the flow field in 3D space is a more complex problem that requires more complicated algorithms combined with 3D reconstruction software.

### 3D reconstruction

It is important to create a comprehensive 3D view of the target sample using continuous ST data from 2D tissue sections in order to interpret the relationship between gene expression and function. 3D reconstruction can restore the natural morphology of tissues and visually observe gene expression on the whole. With the addition of 3D coordinate information, analysis performed in 2D can be transferred to 3D, such as verifying a series of conclusions obtained via immunofluorescence staining through other omics or anatomy knowledge and identifying co-localized gene sets and functional gene modules in the tissue space by combining 3D coordinates. Furthermore, with time-series slices of ST data, cell migration and tissue development can be more thoroughly studied and explored with a 3D view of the tissues. However, 3D reconstruction for ST data is in the early research stage and is still challenging.

3D reconstruction software for ST data is needed to reach the aforementioned goal. 2D slices can be directly aligned and stacked to the 3D reference of the studied tissue if one is available. For example, the Allen Mouse Brain Common Coordinate Framework (CCFv3) reference brain is a 3D spatial template constructed as a population average of 1675 young adult mouse brains imaged using serial two-photon tomography (STPT) from the Allen Mouse Brain Connectivity Atlas [107]. A mouse whole-brain molecular atlas was created by determining the position of each spot in all sections through image registration in the CCFv3 reference [36]. To obtain the full transcriptome profiles of the entire brain, the researchers captured the RNA expression levels of approximately 15,000 genes from 34,000 spatial probes on 75 coronal tissue slices. 3D references of the human brain, macaque brain, and human development have also been constructed [108], [109], [110]. A 3D image generated from magnetic resonance imaging (MRI) or computed tomography (CT) of tissues can also be used as a 3D reference when a high-resolution reference is unavailable. However, a 3D reference does not exist for most species and tissues, and *de novo* reconstruction of ST data is required [111].

The 3D reconstruction process can be divided into three steps (Figure 5C). The first step is tissue segmentation, which separates the tissue-covered area from the background and can be handled by many programs with manual processing. Registration is next performed to align all slices in spatial coordinates and prepare for reconstruction. This step can be performed by many existing software packages, but high-quality original images with high brightness and contrast are required, with minor differences in the rotation angles and translation deviation since a standard automated program cannot handle large differences. After registration, 3D visualization is then performed to obtain a 3D view of the tissues. It is still a challenge for programs to handle large datasets with ultra-high resolution on local machines [112]. A cloud-based 3D visualization software (https://github.com/google/neuroglancer) that borrows computing power from a remote server may solve this problem, but further customization is needed. Integrated tools for 3D reconstruction of ST data containing the aforementioned three functions are under development. Additionally, images derived from ST data contain more uncertainty and have higher computational demands due to their ultra-high resolution compared to images derived from MRI or CT. However, when the research focus is on the continuity of the tissue area rather than the single-cell level, compressing the data from high to low resolution can be considered for better alignment, and the data can be observed and analyzed at a coarse-grained level. Moreover, 3D reconstruction software should be designed to support data analysis and mining of ST data at spatial locations.

## The multi-omics era of single-cell and spatial omics

With the advancement of multi-omics techniques, data can be integrated in different ways based on common characteristics (anchors) of different multi-omics datasets by different methods (Table 2) [113]. Horizontal integration is used to integrate datasets of the same data modality, *e.g.*, two or more scRNA-seq datasets from different sources. With this approach, genes can act as anchors because they are from the same genome. Harmony [114] first maps the dataset into a low-dimensional space and then finds a cell-specific linear correction function through iterative clustering. scVI [53] uses stochastic optimization and deep neural networks to approximate the distribution of observed expression values and learn a non-linear embedding of the cells that can be used for batch correction. LIGER [59] applies integrative non-negative matrix factorization (iNMF) to distinguish dataset-specific factors from shared factors. These methods can achieve better integration results but require recalculation to incorporate a new dataset and can hardly perform on massive datasets. Recently, online iNMF [115] extended the non-negative matrix factorization approach at the heart of LIGER making it possible for integrating large, diverse, and recently completed single-cell datasets using fixed memory. Vertical integration involves datasets collected from the same cells but different omics layers, such as RNA transcription and chromatin accessibility. For example, Seurat v4 [57] learns the relative utility of each data type in each cell using a “weighted-nearest neighbor” framework to enable the integration of multiple modalities. Other popular tools include Multi-Omics Factor Analysis v2 (MOFA+) [116], which has been described as “a multi-omics generalization” of PCA. Diagonal integration involves molecular measurements of unrelated populations of cells that usually relies on fragile biological assumptions and may fail in given scenarios. Many methods, such as MATCHER [117] and MMD-MA [118], use this strategy. However, defining the input data and validating and interpreting the model output are more challenging. scRNA-seq and single-cell assay for transposase-accessible chromatin using sequencing (scATAC-seq) are particularly useful in understanding the underlying molecular mechanisms. scRNA-seq determines which genes are expressed, while scATAC-seq highlights the corresponding regulatory regions. By integrating these datasets, scientists can identify the *cis*-regulatory elements that act on genes, the transcription factors that may control these elements, and more importantly, when and where they are dynamically regulated by comparing different samples. Exploring gene regulatory networks is important for researchers to understand how cell fate is determined.

**Table 2:** **The widely used subset of methods for three types of multi-omics integration**

**Integration type**	**Method**	**Principle**	**URL**	**Language**	**Release year**
Horizontal integration	Harmony	Iterative clustering in dimensionally reduced space	https://github.com/welch-lab/liger	R	2019
	scVI	Bayesian variational autoencoder with a probabilistic formulation	https://scvi-tools.org	Python	2021
	LIGER with online iNMF	Integrative non-negative matrix factorization and joint clustering + online learning	https://github.com/welch-lab/liger	R	2021
Vertical integration	Seurat v4	Weighted nearest neighbor analysis	https://github.com/satijalab/seurat	R	2021
	MOFA+	Generalization of canonical correlation analysis that builds on the Bayesian group factor analysis framework	https://github.com/bioFAM/MOFA2	Python; R	2020
Diagonal integration	MATCHER	Gaussian process latent variable model	https://github.com/jw156605/MATCHER	Python	2017
	MMD-MA	Embedding measured in different ways into a learned latent space	https://bitbucket.org/noblelab/2019_mmd_wabi/src/master/	Python	2019

Traditional single-cell experiments provide details on thousands of molecules at the expense of location information. The latest technological advances have improved the power of ST to systematically measure the expression levels of all or most genes across the tissue space and have been used to generate biological insights from neuroscience, as well as to investigate a range of disease backgrounds. ST data can be integrated with other multi-omics data, providing an extensible framework for the in-depth understanding of biological processes. Integration of ST and single-cell omics (including scRNA-seq, single-nucleus RNA-seq, and scATAC-seq) is currently a hot spot of spatial multi-omics integration. Integration of ST with single-cell omics can take advantage of both technologies and produce high-resolution maps of cellular subpopulations in tissue. This integration can be applied to establish cell-type proportions (for low-resolution ST data) or get cell-type annotation (for high-resolution ST data) of spatial barcoding capture spots, or to get the spatial location of single-cell data by mapping it onto the ST data [13]. Methods like SPOTlight [76], Cell2location [63], and CellTrek [79] are designed to reach that goal. These methods are described in the “Single cell- and tissue-level annotations in ST data” section. With the maturity of spatial multi-omics technology — examples include spatial proteomics technology and spatial chromatin accessibility technology — novel methods for parsing spatial and single-cell omics integration will be needed to be developed accordingly.

However, there are still challenges with the integration of single-cell and spatial omics data that should be settled. The first challenge is to develop additional algorithms and to determine the anchors that support spatial omics integration for different analysis modules. As mentioned in the previous section, multi-omics data can be integrated into different analysis tasks, but the corresponding tools require higher analytical capabilities. The second challenge is to score and rank the features extracted from multiple omics data when the available evidence for the same event is inconsistent. For example, both the transcriptome and proteome can reveal the gene expression, but they are generally not identical. The third challenge is how to make use of the multi-omics data to obtain a macro interpretation of the data at the spatial level after integrating the omics data. The difficult aspect of these challenges is that they are a composite superposition of problems on multiple levels. Resolving these challenges in multi-omics integration requires extensive accumulation of knowledge, a full understanding of the data, and greater technical capabilities for algorithm implementation.

## Future perspectives

Thorough interpretation of ST data requires the development of new technologies and bioinformatics algorithms. This review highlights five topics and the corresponding challenges related to ST data processing. Efficient compression algorithms and parallel computing have been developed to tackle the big data challenge. Algorithms designed to screen data and improve data quality are expected to counteract technical noise or bias in sequencing depth and eliminate the batch effect. Methods for cell identification and annotation can then be applied to define the cell boundary, cluster, and corresponding RNA expression. Tissue-wide data mining approaches enable researchers to investigate the tissue-wide associations of cells and genes in multi-dimensional space. Spatial multi-omics integration provides an extensible framework for the in-depth understanding of biological processes. These next paragraphs discuss the problems of existing algorithms and the future directions for ST development.

Numerous algorithms were developed to address data problems related to defects in the current experimental techniques. For example, most ST techniques cannot accurately locate the cell boundary, and cell identification algorithms have been designed to compensate. Resolution-enhancement algorithms were developed to solve the low-resolution problem. With the development of high-resolution ST technology, algorithms are required to deal with the sparseness of gene expression caused by low capture efficiency. The effect of 3D reconstruction is highly dependent on the data quality. A well-designed technology for ST experiments will have high-quality expression data and well-formatted imaging data to avoid unnecessary pre- and post-processing for downstream analyses. With the development of experimental approaches, data with higher quality will be produced, and the performance of the algorithms will improve accordingly. Similarly, the demands for dedicated algorithms will continue to grow.

Spatial-resolved approaches that consider spatial location and histological information are urgently needed to meet computing requirements. Knowing spatial information ahead of time can improve the prediction accuracy of an algorithm, especially for the prediction tasks of clustering, CCI, and RNA velocity. Histological images can provide algorithms with references for tissue zones and integrity, which is significant in data normalization as well as in identifying spatial domains and SVGs and so on. On the other hand, the huge difference in data distribution and concentration between ST data and single-cell data indicates that algorithms developed for single-cell data may not be suitable for ST data. Along with ST-specific algorithms, corresponding analysis standards should be further established. Unified and high-quality data will be generated through the standard processing workflows for comparison, verification, and integration, which is an essential basis to confirm previous results and explore new biological mechanisms.

Another area that warrants attention is the performance of ST-specific algorithms. The development of ST technologies is in an expansion phase, and various experimental techniques are producing great amounts of data, but a unified approach is lacking. Since ground truth data are almost non-existent, the performance of algorithms can only be judged by comparing the results using common cognitive sense before and after the algorithm is applied. Supervised machine learning algorithms, including supervised neural network algorithms, cannot be trained properly due to the small amount of labeled data available. In addition, the lack of ground truth data also leads to a lack of benchmark evaluation of the algorithms. These problems may be solved with the development and maturity of ST technologies, but remain enormous challenges at present.

Although the technologies and corresponding algorithms still require a long time to develop, researchers have applied ST methods to elucidate the inner workings of discrete cell subpopulations within various contexts and organ systems. These include embryonic development [12], hematopoietic stem cell development and metastasis [18], compilation of a tissue spatial transcriptomic atlas [19], [20], and study of the spatial heterogeneity of diseases, as well as tumor development and tissue microenvironment [21], [119]. These techniques have helped uncover the mechanisms of previously opaque cell–cell communication and biological functions, define disease subtypes, guide prognosis, and target cell populations for precision treatment. For example, multi-dimensional information provided by the ST technologies, such as the location of immune cell infiltration, the type and state of infiltration, and the positional relationship between immune cells and tumor cells, can be comprehensively considered to evaluate the immune infiltration state of the patient. Programmed cell death protein 1 (PD-1) and cytotoxic T lymphocyte-associated protein 4 (CTLA-4) immunotherapies have led to significant progress in tumor treatment [120], [121], [122], [123], but PD-1 immunotherapy is ineffective for a large number of programmed death-ligand 1 (PD-L1)-positive patients. Development of effective therapies requires comprehensive assessment of the spatial information and distribution of specific cell communities for immune checkpoint molecules represented by PD-L1 [124], [125], which may promote the development of measurement and scoring algorithms for new immune checkpoint blockers or new companion diagnostic methods. As the development of ST technologies which moves closer to clinical applications, the corresponding algorithm development will enable ST to solve more difficult problems, including disease prevention, detection, and treatment.

## Competing interests

All authors are current employees of BGI Group Ltd.

## CRediT authorship contribution statement

**Shuangsang Fang:** Conceptualization, Investigation, Writing – review & editing. **Bichao Chen:** Investigation, Writing – review & editing. **Yong Zhang:** Investigation, Writing – original draft. **Haixi Sun:** Investigation, Writing – original draft. **Longqi Liu:** Investigation. **Shiping Liu:** Investigation. **Yuxiang Li:** Supervision, Conceptualization. **Xun Xu:** Supervision, Conceptualization. All authors have read and approved the final manuscript.
